# Cholesterol crystal embolism in multiple organs after transarterial chemoembolization for hepatocellular carcinoma: An autopsy case report

**DOI:** 10.1097/MD.0000000000030769

**Published:** 2022-09-30

**Authors:** Junki Yamashita, Takuto Nosaka, Kazuto Takahashi, Tatsushi Naito, Kazuya Ofuji, Hidetaka Matsuda, Masahiro Ohtani, Katsushi Hiramatsu, Motohiro Kobayashi, Yasunari Nakamoto

**Affiliations:** a Second Department of Internal Medicine, Faculty of Medical Sciences, University of Fukui, Fukui, Japan; b Department of Tumor Pathology, Faculty of Medical Sciences, University of Fukui, Fukui, Japan.

**Keywords:** autopsy, cholesterol crystal embolism, hepatocellular carcinoma, transcatheter arterial chemoembolization

## Abstract

Transcatheter arterial chemoembolization (TACE) for hepatocellular carcinoma (HCC) rarely causes cholesterol crystal embolism (CCE). In our case, the histological findings suggested that the onset of CCE occurred at different time points in different organs.

**Patient concerns:**

A 72-year-old Japanese woman with HCC underwent TACE. After TACE, serum creatinine level and eosinophil count gradually increased. Three months later, she was admitted to our department with a fever and back pain.

**Diagnosis:**

Laboratory examinations showed sepsis with disseminated intravascular coagulation. She was treated with antimicrobial agents and anticoagulants, but died of multiple organ failure.

**Interventions:**

An autopsy was performed to examine the cause of multiple organ failure after 3 months of TACE.

**Outcomes:**

A mixture of both chronic phase emboli with intimal thickening and fibrosis and acute phase emboli with inflammatory cell infiltration were observed in the small intestine. Moreover, multiple intravascular cholesterol fissures were observed in the kidney, stomach, duodenum, colon, pancreas, and spleen, which were the vascular dominant organs of the celiac artery and superior mesenteric artery. These histological findings suggested that cholesterol crystals were continuously disseminated after TACE.

**Lessons:**

TACE for HCC may cause progressive CCE and damage in multiple organs. When progressive renal dysfunction, eosinophilia, or multiple organ dysfunction is observed after TACE, the CCE should be suspected.

## 1. Introduction

Cholesterol crystal embolism (CCE) is caused by dissemination of cholesterol crystals from a disrupted atherosclerotic plaque. Approximately 80% of cases are caused by iatrogenic factors such as endovascular manipulation and anticoagulation therapy.^[1]^ However, transcatheter arterial chemoembolization (TACE) rarely causes CCE.^[2]^

## 2. Case report

**Figure 1.**
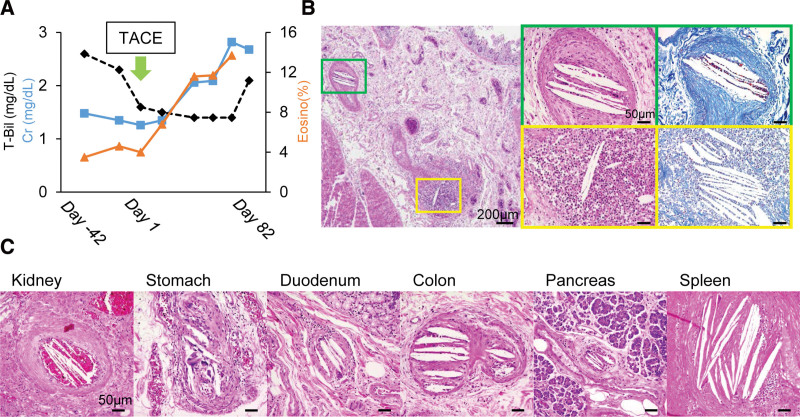
(A) Clinical course of the patient. The transition of creatinine (Cr), eosinophil percentage (Eosino), and total bilirubin (T-bil) from 42 days before to 82 days after TACE. (B) HE staining of the small intestine specimen shows CCE. Magnified images in the green square and yellow square (left, HE staining; right, Azan staining). (C) HE stained specimens of the kidney, stomach, duodenum, colon, pancreas, and spleen show CCE. CCE = cholesterol crystal embolism, HE = Haematoxylin/eosin, TACE = transcatheter arterial chemoembolization.

A 72-year-old Japanese woman was referred to our department for treatment of hepatocellular carcinoma (HCC). Her medical history included liver cirrhosis (type C), angina pectoris, and diabetes mellitus. Transcatheter arterial chemoembolization (TACE) was performed for a 28-mm HCC in the liver (S7). After TACE, the creatinine level and eosinophil count increased, but no increase in bilirubin levels was noted (Fig. 1A). Three months after TACE, the patient presented to the emergency department with a fever and back pain. The laboratory examinations were shown in Table 1. The patient was diagnosed with sepsis with disseminated intravascular coagulation and was treated with antimicrobial agents and anticoagulants. However, she died of multiple organ failure, and an autopsy was performed. Histologically, intravascular cholesterol fissures suggestive of CCE were noted. Histopathologically, the small intestine showed a mixture of chronic lesions, such as narrowing of the vessel lumen due to intimal thickening and fibrosis (Fig. 1B; green square), and acute lesions such as infiltration of inflammatory cells into the vessel wall (Fig. 1B; yellow square). Multiple intravascular cholesterol fissures were also observed in the kidneys, stomach, duodenum, colon, pancreas, and spleen (Fig. 1C).

**Table 1:** Laboratory examinations.

WBC	7,400	/µL	Na	128	mmol/L
Neutro	93	%	Cl	105	mmol/L
Eosino	1.0	%	K	4.5	mmol/L
Baso	0.0	%	Ca	7.3	mg/dL
Lymph	3.0	%	UA	6.6	mg/dL
Mono	3.0	%	BUN	42	mg/dL
RBC	255 × 10^4^	/µL	Cre	2.58	mg/dL
Hb	7.8	g/dL	TP	5.8	g/dL
Plt	7.1 × 10^4^	/µL	Alb	2.6	g/dL
			T-bil	1.6	mg/dL
FDP	53.8	µg/mL	AST	78	U/L
PT	49.2	%	ALT	37	U/L
PT-INR	1.54		LDH	274	U/L
D-dimer	25.2	µg/mL	ALP	162	U/L
			γ-GTP	23	U/L
CRP	6.68	mg/dL	ChE	37	U/L
PCT	8.44	ng/mL	Amy	62	U/L

γ-GTP = gamma-glutamyl transpeptidase, Alb = albumin, ALP = alkaline phosphatase, ALT = alanine transaminase, Amy = amylase, AST = aspartate transaminase, BUN = blood urea nitrogen, ChE = cholinesterase, Cre = creatinine, CRP = C-reactive protein, FDP = fibrin/fibrinogen degradation products, Hb = hemoglobin, INR = international normalized ratio, LDH = lactate dehydrogenase, PCT = procalcitonin, Plt = platelets, PT = prothrombin time, RBC = red blood cells, T-bil = total bilirubin, TP = total protein, UA = uric acid, WBC = white blood cells.

## 3. Discussion

Risk factors for CCE include a history of atherosclerotic vascular disease and general risk factors for atherosclerosis.^[1,3]^ CCE is characterized by cutaneous symptoms, renal dysfunction, gastrointestinal symptoms, and eosinophilia.^[1,3,4]^ There is no curative treatment for CCE, and the mainstay of treatment is supportive care of organs exhibiting embolic symptoms and prevention of recurrence.^[1,3]^ Hamura et al^[2]^ reported a case of CCE that developed after TACE, which resulted in small bowel perforation, and speculated that emboli originating from the celiac artery may have dispersed into the superior mesenteric artery (SMA) via the pancreaticoduodenal artery arcade, or that atheroma may have existed in the main trunk of the SMA. In our case, TACE for HCC caused CCE, and findings of CCE were observed in multiple organs. Before TACE and at the time of autopsy, severe atherosclerosis with calcification was observed in the aorta, suggesting that TACE induced CCE in organs distributed by the celiac artery and SMA.

Renal dysfunction due to CCE occurs in 3 forms: acute, subacute, and chronic.^[1,4]^ Acute renal dysfunction occurs within 1 week after induction by dissemination of large amounts of cholesterol crystals and accounts for 20% to 30% of cases of renal dysfunction due to CCE.^[1]^ Subacute renal dysfunction is the most common, with persistent or recurrent small emboli that produce progressive renal dysfunction within weeks to months after induction.^[1,4]^ Chronic renal dysfunction is difficult to distinguish from nephrosclerosis and ischemic nephropathy.^[1,4]^ Ito et al^[5]^ reported the autopsy findings in 3 cases with acute, subacute, and chronic clinical courses, respectively, after cardiovascular procedures. In our case, progressive renal dysfunction was observed approximately 3 months after TACE. The histological findings suggested that the onset of CCE occurred at different time points in different organs. Thus, it was suggested that cholesterol crystals were continuously disseminated after TACE.

TACE is a rare cause of CCE, and there are few reports of endovasucular treatment for HCC causing CCE (Table 2). We presented a rare case of histologically confirmed CCE in multiple organs after TACE for HCC. When progressive renal dysfunction, eosinophilia, or multiple organ dysfunction is observed after TACE, the CCE should be suspected.

**Table 2:** Previous reports of cholesterol crystal embolism during endovascular treatment for hepatocellular carcinoma

Author	Year	Age	Sex	Endovascular treatment	Symptoms	Time to onset	Diagnostic modality	Embolized organs	Outcome	Reference
Yamanishi et al.	2014	70s	M	Reservoir implantation for HAIC	Numbness and pain in the legs	1 month	Autopsy	Legs	Dead	NA
Hamura et al	2021	71	M	TACE	Abdominal pain, Fever	5 days	Laparotomy	Small bowel	Alive	^[2]^
Our case	2018	72	F	TACE	Abdominal pain, Renal dysfunction	2.7 months	Autopsy	Stomach, Small bowel, Colon, Kidney, Spleen, Pancreas	Dead	

70s = seventies, F = female, HAIC = hepatic arterial infusion chemotherapy, M = male, NA = not applicable, TACE = transcatheter arterial chemoembolization.

## Acknowledgements

The authors would like to thank Editage for the English language editing.

## Author contributions

**Supervision:** Yasunari Nakamoto.

**Writing** – original draft: Junki Yamashita.

**Writing** – review & editing: Takuto Nosaka, Kazuto Takahashi, Tatsushi Naito, Kazuya Ofuji, Hidetaka Matsuda, Masahiro Ohtani, Katsushi Hiramatsu, Motohiro Kobayashi, Yasunari Nakamoto.
